# Exposure to Secondhand Smoke Among Nonsmokers — United States, 1988–2014

**DOI:** 10.15585/mmwr.mm6748a3

**Published:** 2018-12-07

**Authors:** James Tsai, David M. Homa, Andrea S. Gentzke, Margaret Mahoney, Saida R. Sharapova, Connie S. Sosnoff, Kevin T. Caron, Lanqing Wang, Paul C. Melstrom, Katrina F. Trivers

**Affiliations:** ^1^Office on Smoking and Health, National Center for Chronic Disease Prevention and Health Promotion, CDC; ^2^Division of Laboratory Sciences, National Center for Environmental Health, CDC.

## Abstract

Exposure to secondhand smoke from burning tobacco products can cause sudden infant death syndrome, respiratory infections, ear infections, and asthma attacks in infants and children, and coronary heart disease, stroke, and lung cancer in adult nonsmokers (*1*). There is no risk-free level of secondhand smoke exposure (*2*). CDC analyzed questionnaire and laboratory data from the National Health and Nutrition Examination Survey (NHANES) to assess patterns of secondhand smoke exposure among U.S. nonsmokers. The prevalence of secondhand smoke exposure among U.S. nonsmokers declined substantially during 1988–2014, from 87.5% to 25.2%. However, no change in exposure occurred between 2011–2012 and 2013–2014, and an estimated one in four nonsmokers, or approximately 58 million persons, were still exposed to secondhand smoke during 2013–2014. Moreover, marked disparities persisted across population groups. Exposure prevalence was highest among nonsmokers aged 3–11 years (37.9%), non-Hispanic blacks (50.3%), and those who were living in poverty (47.9%), in rental housing (38.6%), or with someone who smoked inside the home (73.0%), or among persons who had less than a high school education (30.7%). Comprehensive smoke-free laws and policies for workplaces and public places and smoke-free rules for homes and vehicles can further reduce secondhand smoke exposure among all nonsmokers.

NHANES is a program of studies designed to assess the health and nutritional status of children and adults in the United States (*3*). Participants are recruited using a household-based, multistage, stratified sampling scheme designed to represent the noninstitutionalized civilian U.S. population (*3*).* NHANES includes a home interview and physical examination at a mobile examination center where biologic specimens are collected for laboratory testing, including serum cotinine, an indicator of recent nicotine exposure (*4*,*5*).^†^ Questionnaire and laboratory data were collected from participants (or their guardians) aged ≥4 years during NHANES III 1988–1994 and aged ≥3 years during biennial NHANES 1999–2014. Interview response rates ranged from 71.0% (2013–2014) to 86.0% (1988–1994). Response rates for mobile examination center samples ranged from 68.5% (2013–2014) to 80.0% (2001–2002) (*3*). An established standard range of serum cotinine of 0.05–10 ng/mL was used to define secondhand smoke exposure among nonsmokers and to allow for historical comparisons (*6*,*7*).^§^ Nonsmokers were defined as 1) children aged 4–11 years (NHANES III 1988–1994) and children aged 3–11 years (NHANES 1999–2014) with serum cotinine ≤10 ng/mL; 2) adolescents aged 12–19 years with serum cotinine ≤10 ng/mL and who did not report smoking within the preceding 30 days or use of any nicotine-containing product within the preceding 5 days at mobile examination center interview; and 3) adults aged ≥20 years with serum cotinine ≤10 ng/mL and who did not report being a current smoker during household interview or use of any nicotine-containing product within the preceding 5 days at mobile examination center interview.^¶^

To assess prevalence of secondhand smoke exposure during 1988–2014, the percentage of persons with serum cotinine levels 0.05–10 ng/mL for each survey cycle was calculated among nonsmokers overall by age group (3–11, 12–19, and ≥20 years), and, among children aged 3–11 years, by race and Hispanic origin** (non-Hispanic white, non-Hispanic black, or Mexican American). During 2013–2014, percentages and 95% confidence intervals of secondhand smoke exposure were computed among nonsmokers by age, sex, race and Hispanic origin, poverty, education, housing status, and whether the participant lived with someone who smoked inside the home. Percentage differences within each subgroup were assessed using chi-squared tests, with statistical significance defined as p<0.05. Estimated numbers of persons exposed to secondhand smoke during 2013–2014 were calculated according to population estimates from the American Community Survey.^††^ Data were weighted using examination sample weights to account for the complex survey design and differential probability of sample selection, nonresponse, and noncoverage.

From 1988–1991 to 2013–2014, the prevalence of secondhand smoke exposure declined 71.2% among U.S. nonsmokers, from 87.5% to 25.2%. Secondhand smoke exposure declined from 87.8% to 37.9% among children aged 3–11 years (56.8% decrease), from 87.4% to 32.0% among adolescents aged 12–19 years (63.4% decrease), and from 87.4% to 22.0% among adults aged ≥20 years (74.8% decrease) (Figure 1). Among nonsmokers aged 3–11 years, secondhand smoke exposure declined from 86.4% to 37.8% among non-Hispanic whites (56.3% decrease), from 94.5% to 66.1% among non-Hispanic blacks (30.1% decrease), and from 84.4% to 22.2% among Mexican Americans (73.7% decrease) (Figure 2). From 2011–2012 to 2013–2014, no statistically significant change occurred in the prevalence of secondhand smoke exposure among U.S. nonsmokers.

**FIGURE 1:**
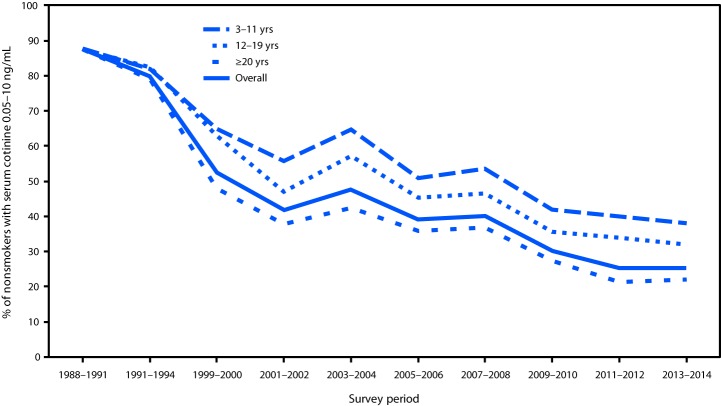
Percentage of nonsmokers aged ≥3 years* with evidence of secondhand smoke exposure (serum cotinine levels 0.05–10 ng/mL), by age group — National Health and Nutrition Examination Survey (NHANES), United States, 1988–2014 * Nonsmokers aged ≥4 years for NHANES III 1988–1994.

**FIGURE 2:**
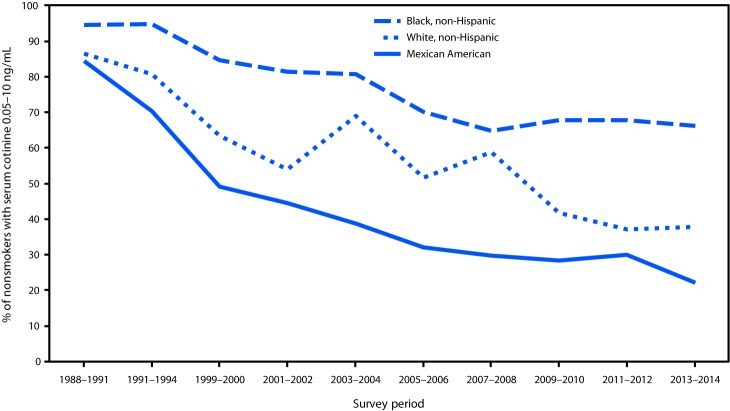
Percentage of nonsmokers aged 3–11 years* with evidence of secondhand smoke exposure (serum cotinine levels 0.05–10 ng/mL), by race and ethnicity^†^— National Health and Nutrition Examination Survey (NHANES), United States, 1988–2014 * Nonsmokers aged ≥4 years for NHANES III 1988–1994. ^†^ Because of sample design, racial and Hispanic origin categories were limited to non-Hispanic whites, non-Hispanic blacks, and Mexican Americans across all survey cycles.

During 2013–2014, the prevalence of secondhand smoke exposure was significantly higher among children aged 3–11 years (37.9%) than among adults aged ≥20 years (22.0%) (Table), among non-Hispanic blacks (50.3%) than among non-Hispanic whites (21.4%) and Mexican Americans (20.0%), and among persons living below the poverty level (47.9%) compared with those living at or above the poverty level (21.2%). By education, among persons aged ≥25 years, the prevalence of secondhand smoke exposure was highest among those with less than a high school education (30.7%) and lowest among those with a college degree or higher (10.8%). The prevalence of secondhand smoke exposure was significantly higher among persons who rented (38.6%) than among those who owned their homes (19.2%). In addition, the prevalence among persons who lived with anyone who smoked inside the home (73.0%) was significantly higher than it was among those who did not (22.3%).

**TABLE:** Percentage of nonsmokers aged ≥3 years with serum cotinine levels 0.05–10 ng/mL, by selected sociodemographic characteristics — National Health and Nutrition Examination Survey, United States, 2013–2014

Characteristic	% (95% CI)
**Overall**	25.2 (21.1–29.8)
**Sex**	
Male	27.1 (23.0–31.6)
Female	23.6 (19.0–28.9)
**Age group (yrs)**	
3–11	37.9 (31.2–45.0)
12–19	32.0 (24.9–39.9)
≥20	22.0 (18.4–26.1)
Race and Hispanic origin*	
White, non-Hispanic	21.4 (16.1–27.8)
Black, non-Hispanic	50.3 (44.8–55.8)
Mexican American	20.0 (16.1–24.6)
**Poverty status**	
Below poverty level^†^	47.9 (42.2–53.7)
At or above poverty level	21.2 (17.4–25.7)
Unspecified	23.3 (17.6–30.1)
**Education^§^**	
Less than high school diploma	30.7 (25.4–36.5)
High school diploma or equivalent	28.8 (21.7–37.0)
Some college or associate’s degree	23.5 (19.2–28.5)
Bachelor’s degree or higher	10.8 (8.1–14.3)
**Housing**	
Own	19.2 (15.0–24.3)
Rent	38.6 (33.9–43.5)
Other arrangement	35.9 (22.1–52.5)
**Living with anyone who smoked inside the home**	
Yes	73.0 (59.2–83.4)
No	22.3 (18.7–26.5)

**Abbreviation:** CI = confidence interval.

* Data by race and Hispanic origin were limited to the three racial and Hispanic origin groups available across all survey cycles (non-Hispanic white, non-Hispanic black, and Mexican American).

^†^ Income-to-poverty ratio <1.0.

^§^ Assessed for persons aged ≥25 years.

Among the estimated 58.0 million nonsmokers who were exposed to secondhand smoke during 2013–2014, approximately 36.7 million were adults, 9.1 million were adolescents, and 14.0 million were children.^§§^ This includes 6.8 million non-Hispanic whites, 3.3 million non-Hispanic blacks, and 1.5 million Mexican Americans.^¶¶^

## Discussion

Although secondhand smoke exposure among U.S. nonsmokers declined from 87.5% to 25.2% during 1988–2014, progress has stalled in recent years. One in four nonsmokers were still exposed to secondhand smoke during 2013–2014, and disparities in exposure prevalence persisted across demographic groups. Prevalence remained highest among children aged 3–11 years, non-Hispanic blacks, and persons living in poverty, in rental housing, and with someone who smoked inside the home. Enhanced and equitable implementation of comprehensive smoke-free laws and policies for workplaces and public places and smoke-free rules for homes and vehicles can further reduce secondhand smoke exposure among all nonsmokers (*2*).***

The decline in secondhand smoke exposure among U.S. nonsmokers is likely due to decreasing cigarette smoking rates, increased awareness of the risks for secondhand smoke exposure, and the adoption of comprehensive smoke-free laws prohibiting smoking in workplaces and public places in many states and localities (*1*,*8*,*9*). During 2011–2014, the percentage of nonsmokers exposed to secondhand smoke did not decline significantly across most demographic subgroups (*6*). This lack of decline could be attributable to slowed adoption of statewide comprehensive smoke-free laws during this period (*10*). Nonetheless, to date, 27 states and the District of Columbia have comprehensive smoke-free laws, and progress in smoke-free law adoption has occurred at the local level in more recent years.^†††^ Moreover, during 2015–2017, 199 localities adopted comprehensive smoke-free laws, and 21 additional localities have implemented such laws as of July 2018.^§§§^ In addition, the U.S. Department of Housing and Urban Development adopted a rule requiring most public housing to be smoke-free by July 31, 2018, and Alaska adopted a statewide law in 2018 prohibiting smoking in workplaces and public places, although localities can opt out.^¶¶¶^

Disparities in secondhand smoke exposure persisted, with higher exposure among children aged 3–11 years (37.9%) and non-Hispanic blacks (50.3%) than among other age or racial and Hispanic origin subgroups. Variations in smoking prevalence, smoke-free policy coverage, and knowledge about the harms of secondhand smoke might have contributed to these disparities. These findings underscore the importance of continued measures to enhance smoke-free policy coverage, including educating parents and caregivers about the benefits of voluntarily prohibiting smoking in their homes and vehicles. These steps can reduce secondhand smoke exposure across all population groups, particularly those with the greatest exposure prevalence.

The findings in this report are subject to at least five limitations. First, smoking status was based on self-report and could be subject to social desirability and reporting biases. Some smokers might misrepresent their smoking status in surveys. Second, serum cotinine levels reflect recent exposure; thus, exposure misclassification might have occurred. Third, an established standard range of serum cotinine was used to define secondhand smoke exposure, which allowed historical comparisons. However, secondhand smoke exposure below this cutpoint might not have been measured. Fourth, serum cotinine might reflect secondhand exposure to other tobacco products such as e-cigarettes, which was not assessed in the survey. Finally, sample design limited the racial and Hispanic populations that could be assessed.

Although secondhand smoke exposure among U.S. nonsmokers has decreased considerably during the past two and a half decades, progress has stalled in recent years, and approximately one in four nonsmokers remains exposed to this preventable health hazard. In addition, disparities persist: 14.0 million children aged 3–11 years, including two of every three non-Hispanic black children, were still exposed during 2013–2014. Continued measures to implement comprehensive smoke-free laws in workplaces and public places, adoption of smoke-free home and vehicle rules, and educational interventions warning about the risks for secondhand smoke exposure can further reduce secondhand smoke exposure, especially among vulnerable populations.

**Summary**

**What is already known about this topic?**

Exposure to secondhand tobacco smoke can cause sudden infant death syndrome, respiratory infections, ear infections, and asthma attacks in infants and children, and coronary heart disease, stroke, and lung cancer in adult nonsmokers.

**What is added by this report?**

Although secondhand smoke exposure among U.S. nonsmokers declined from 87.5% to 25.2% during 1988–2014, one in four nonsmokers, including 14 million children, were exposed to secondhand smoke during 2013–2014.

**What are the implications for public health practice?**

Continued measures to implement comprehensive smoke-free laws in workplaces and public places, adoption of smoke-free home and vehicle rules, and educational interventions warning about the risks for secondhand smoke exposure can further reduce secondhand smoke exposure.

## Footnotes

* Stand-alone NHANES were conducted in 1976–1980 and 1988–1994 (Phase 1: 1988–1991; Phase 2: 1991–1994); since 1999, NHANES has been a continuous study conducted for 2-year periods. https://www.cdc.gov/nchs/nhanes/index.htm.

† Serum cotinine is based on blood samples collected by venipuncture. Since 1988, data are collected and laboratory analyses are performed using an isotope dilution liquid chromatography tandem mass spectrometry method. Laboratory analytic and quality assurance and quality control procedures are described in detail. https://wwwn.cdc.gov/nchs/data/nhanes/2013-2014/manuals/2013_MEC_Laboratory_Procedures_Manual.pdf.

§ During 1988–2000, the laboratory limit of detection for serum cotinine was 0.05 ng/mL. Since 2001–2002, the laboratory limit of detection for serum cotinine has been 0.015 ng/mL.

¶ Based on response to the question “During the past 5 days, did (you/he/she) use any product containing nicotine including cigarettes, pipes, cigars, chewing tobacco, snuff, nicotine patches, nicotine gum, or any other product containing nicotine?” For 2013–2014, questions assessed included “During the past 5 days, including today, did you smoke cigarettes, pipes, cigars, little cigars or cigarillos, water pipes, hookahs, or e-cigarettes?” “During the past 5 days, including today, did (you/he/she) use any smokeless tobacco (chewing tobacco, snuff, snus, or dissolvables)?” and “During the past 5 days, including today, did you use any nicotine replacement therapy products such as nicotine patches, gum, lozenges, inhalers, or nasal sprays?” Serum cotinine concentrations >10 ng/mL are associated with recent active smoking.

** Because of the NHANES sample design, only data for non-Hispanic whites, non-Hispanic blacks, and Mexican Americans are available across all survey cycles. However, all racial and ethnic groups are included in the reported estimates for the total population and subgroups by sex, age, poverty, education, housing, and status of living with anyone who smoked inside the home.

††
https://wwwn.cdc.gov/nchs/data/nhanes3/ResponseRates/2013-2014_ACS_Control_Total.xlsx .

§§ Numbers do not sum to total because of rounding.

¶¶ Children of all racial ethnic groups are included in the total population estimate.

*** https://www.thecommunityguide.org/search/secondhand.

††† https://chronicdata.cdc.gov/Legislation/STATE-System-Smokefree-Indoor-Air-Fact-Sheet/vgq2-kkcg.

§§§ https://no-smoke.org/wp-content/uploads/pdf/EffectivePopulationList.pdf.

¶¶¶ https://www.gpo.gov/fdsys/pkg/FR-2016-12-05/pdf/2016-28986.pdf; http://www.akleg.gov/basis/Bill/Text/30?Hsid=SB0063F.
